# Prevalence and risk of deep vein thrombosis recurrence in Thai patients with iliac compression syndrome

**DOI:** 10.1016/j.rpth.2026.103348

**Published:** 2026-01-13

**Authors:** Hathamon Chonwarangkoon, Chinnarat Bua-Ngam, Sasiprapa Rongthong, Pichika Chantrathammachart, Pimjai Niparuck, Teeraya Puavilai, Thanakrit Piyajaroenkij, Pantep Angchaisuksiri, Kochawan Boonyawat

**Affiliations:** 1 Department of Medicine, Hemostasis and Thrombosis Center, Faculty of Medicine, Ramathibodi Hospital, Mahidol University, Bangkok, Thailand; 2 Department of Diagnostic and Therapeutic Radiology, Faculty of Medicine, Ramathibodi Hospital, Mahidol University, Bangkok, Thailand; 3 Department of Medicine, Faculty of Medicine, Ramathibodi Hospital, Mahidol University, Bangkok, Thailand

**Keywords:** deep vein thrombosis, iliac compression syndrome, May–Thurner syndrome, postthrombotic syndrome, pulmonary embolism, venous thromboembolism

## Abstract

**Background:**

Iliac compression syndrome (ICS) results from compression of the left common iliac vein by the overlying right common iliac artery. The true prevalence of ICS remains unknown.

**Objectives:**

To study the prevalence of ICS in patients with deep vein thrombosis (DVT) of the legs and to evaluate outcomes, including recurrent DVT, pulmonary embolism, and postthrombotic syndrome, in ICS patients.

**Methods:**

We conducted a retospective study from January 2015 to December 2023. Patients objectively confirmed diagnosis of DVT who underwent imaging either CT or MRI of the lower abdomen were included. The imaging studies were reviewed by 2 radiologists.

**Results:**

Among the 180 patients included, the prevalence of ICS was 37.2% (67/180). Left leg DVT was more frequent in ICS patients than in non-ICS patients (62.7% vs 23.9%; *P* = .01). There was no significant difference in iliac vein involvement (34.3% vs 22.1%; *P* = .07), pulmonary embolism (13.4% vs 20.4%; odds ratio [OR], 0.61; 95% CI, 0.3-1.4; *P* = .24), or postthrombotic syndrome (4.5% vs 1.8%; OR, 2.60; 95% CI, 0.4-16.0; *P* = .30). Recurrent DVT was more frequent in ICS patients (17.9% vs 4.4%; OR, 4.71; 95% CI, 1.6-14.1; *P* = .003). Factors significantly associated with recurrent DVT were ICS (OR, 8.26; 95% CI, 2.03-33.66; *P* = .003), previous venous thromboembolism (OR, 34.29; 95% CI, 7.38-159.1; *P* < .001), and autoimmune diseases (OR, 6.31; 95% CI, 1.47-26.96; *P* = .01).

**Conclusion:**

The prevalence of ICS in DVT patients in this study was 37%. ICS was associated with an increased risk of recurrent DVT. ICS might be an underinvestigated contributor to recurrent DVT and warrants further exploration.

## Essentials

• Prevalence and clinical consequences of iliac compression syndrome (ICS) remain unclear.
• ICS was identified by abdominal CT or MRI imaging in DVT patients.
• ICS prevalence was 37% with younger age significantly associated with its presence.
• Patients with ICS had significantly higher recurrent DVT which suggests an under investigated contributor.

## 1 Introduction

Iliac compression syndrome (ICS), also known as May–Thurner syndrome, is an anatomical variant in which the right common iliac artery compresses the left common iliac vein (CIV) against the lumbar spine, predisposing to left-sided deep vein thrombosis (DVT). This chronic pulsatile compression leads to intimal fibrosis and spur formation, promoting thrombosis [1]. Previous studies have reported iliac vein compression in 61.3% to 76.1% of patients with left iliofemoral DVT [2]. While anatomical compression is found in 14% to 32% of autopsy cases, clinically apparent ICS accounts for only 2% to 5% of all DVTs [1]. This discrepancy is likely due to the often asymptomatic nature of ICS and the limitations of standard diagnostic methods. Venography combined with intravascular ultrasound is considered the gold standard for diagnosis, but these methods are invasive, resource-intensive, and not routinely performed [1]. Noninvasive imaging modalities, such as computed tomography (CT) venography and magnetic resonance (MR) venography, have emerged as valuable alternatives, offering high sensitivity and specificity in detecting iliac vein compression. They also help exclude other causes of compression, such as lymphadenopathy, tumor, or hematoma [3,4].

Management of ICS typically involves anticoagulation and, in select cases, catheter-directed thrombolysis with stenting to relieve both thrombus burden and mechanical obstruction [1]. Anticoagulation alone may be insufficient, particularly in patients with extensive iliofemoral DVT or recurrent symptoms. Postthrombotic syndrome (PTS) develops in 20% to 50% of DVT cases, increasing to 70% in patients with ICS [5]. In contrast, pulmonary embolism (PE) appears to be less common in ICS-related DVT [6].

Despite these observations, the prevalence of ICS and its impact on recurrent DVT remain poorly defined. Therefore, this study evaluated the prevalence of ICS among Thai patients with DVT and its association with clinical outcomes, including recurrent DVT, PE, and PTS. Our aim was to raise awareness of ICS and to explore its potential implications for patient management and prognosis.

## 2 Methods

### 2.1 Study design

We conducted a single-center, retrospective cohort study at Ramathibodi Hospital, Mahidol University, Bangkok, Thailand, between January 2015 and December 2023.

Patients were included if all of the following conditions were present: 1) age >18 years, 2) objectively confirmed diagnosis of lower extremity DVT by Doppler ultrasound, CT of the abdomen, or CT venography, and 3) underwent imaging, either a CT scan or an MR imaging (MRI) of the abdomen, either before or after diagnosis of DVT. Patients were excluded if DVT was caused by external compression of the vein by a mass, tumor, or lymphadenopathy.

The imaging studies were reviewed by 2 radiologists. ICS was diagnosed when the left CIV was compressed by the right common iliac artery, and the stenosis of the left CIV exceeded 50% in luminal diameter, with or without evidence of a filling defect or collateral veins [4]. The degree of venous compression was calculated as the diameter of the left CIV at the site of maximal compression divided by the maximal diameter of the uncompressed caudal left CIV.

Ethical approval was granted by the Research Ethics Committee of the Faculty of Medicine, Ramathibodi Hospital, Mahidol University (approval number MURA2023/878).

### 2.2 Data collection

The following information was retrieved from hospital electronic medical records: age, sex, body mass index (BMI), affected leg, symptoms at presentation, date of DVT diagnosis, location of the DVT, ICS diagnostic modality (CT scan or MRI of the abdomen), treatments (deny treatment, low-molecular-weight heparin, vitamin K antagonist, direct oral anticoagulant, inferior vena cava filter, or endovascular stent), and DVT risk factors. The risk factors included obesity, defined as a BMI ≥ 25 kg/m^2^ [7], surgery under general anesthesia for >30 minutes, immobilization for at least 3 days, estrogen use, long-distance travel >4 hours, autoimmune disease, hereditary thrombophilia, active malignancy, lower extremity fracture, and pregnancy or postpartum. Unprovoked DVT was defined as DVT in patients with no identifiable risk factors. DVT outcomes, including recurrent DVT, PE, and PTS, were also recorded using the Villalta score.

### 2.3 Statistical analysis

Sample size was calculated based on a previous study demonstrating a clinically recognized prevalence of ICS of 2% to 5% [1]. An expected prevalence of 4% was calculated in this study. A total of 164 patients were required for this study to obtain a 95% CI with a precision of 0.03.

Descriptive statistics were used to describe patients’ demographic data. Categorical variables were presented as percentages. Continuous variables were presented as means and SDs or medians and IQRs, as appropriate. The prevalence of ICS and the occurrence of outcomes were reported as percentages. Risk factors associated with ICS and outcomes were analyzed using logistic regression. Univariate logistic regression was performed, and variables with a *P* value < .2 were included in the multivariate analysis. Multiple logistic regression was performed using a backward stepwise approach. Odds ratios (ORs) and 95% CIs were calculated. A *P* value of .05 was considered statistically significant. All statistical analyses were performed using STATA version 18.0 (StataCorp LLC).

## 3 Results

### 3.1 Populations

Among 375 patients diagnosed with proximal DVT of the legs, 195 (52%) were excluded. CT or MRI was not performed in 188/375 (50%) patients, as indicated in the inclusion criteria. Seven patients (1.9%) were excluded due to the CIV compressed by a tumor or lymph node. A flow chart is presented in Figure.

**Figure:**
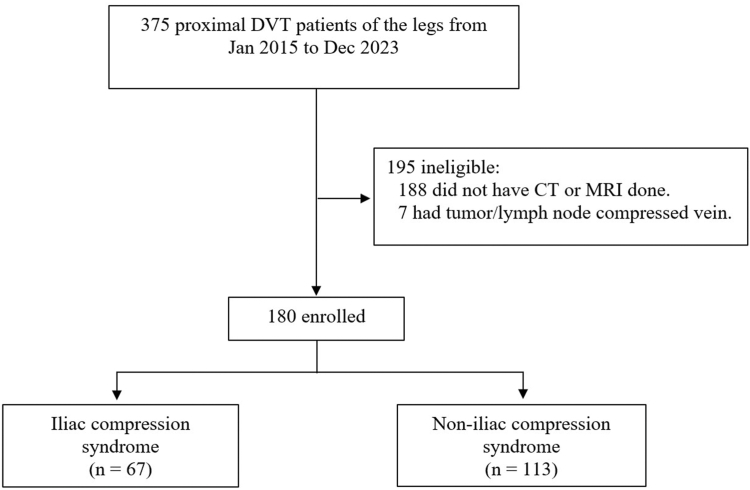
Protocol flow chart. CT, computed tomography; DVT, deep vein thrombosis; MRI, magnetic resonance imaging.

### 3.2 Baseline characteristics

A total of 180 patients were included in this study. The median age across all study groups was 68 years (IQR, 55.5-76); 65% of all patients were female. Half of the DVTs were left leg DVTs (50.6%); most patients presented with leg swelling (77.8%). The most common location of DVTs was the femoropopliteal vein (28.9%), whereas 26.7% of DVT patients had iliac vein involvement. Abdominal CT scan and CT angiography were performed in 72.8% and 16.1% of cases, respectively. Abdominal MRI and MR angiography were performed in 10.6% and 0.6% of cases, respectively. The most common DVT risk factors were immobilization (43.9%); 16.7% of DVTs were unprovoked. Vitamin K antagonists were the mainstay of treatment in both groups (47.2%); low-molecular-weight heparin was the second most common treatment (25.6%). The mean iliac vein compression percentage was 40.7% (SD, 21.4%). Other baseline characteristics are presented in Table 1.

**Table 1:** Baseline characteristics of the 180 patients with deep vein thrombosis.

Variables	All patients (n = 180)	ICS (*n* = 67)	Non-ICS (*n* = 113)	*P* value
Age (y), median (IQR)	68.0 (55.5-76)	58.0 (46-72)	71.0 (61-79)	<.01
Female	117 (65.0)	43 (64.2)	74 (65.5)	.86
Male	63 (35.0)	24 (35.8)	39 (34.5)	
Affected leg				
Left	91 (50.6)	42 (62.7)	49 (43.4)	.01
Right	65 (36.1)	16 (23.9)	49 (43.4)	.01
Both	24 (13.3)	9 (13.4)	15 (13.3)	.98
Symptoms at presentation				
No symptom	26 (14.4)	12 (17.9)	14 (12.4)	.31
Swelling	140 (77.8)	50 (74.6)	90 (79.7)	.43
Pain	6 (3.3)	3 (4.5)	3 (2.7)	.51
Location				
Iliac vein involvement	48 (26.7)	23 (34.3)	25 (22.1)	.07
Femoropopliteal vein	52 (28.9)	16 (23.9)	36 (31.9)	.25
Isolated tibial or peroneal vein	12 (6.7)	6 (9.0)	6 (5.3)	.34
Diagnostic modality				
Abdominal CT scan	131 (72.8)	46 (68.7)	85 (75.2)	.34
CT angiography	29 (16.1)	16 (23.9)	13 (11.5)	.03
Abdominal MRI	19 (10.6)	4 (6.0)	15 (13.3)	.12
MR angiography	1 (0.6)	1 (1.5)	0 (0.0)	.19
Risk factors				
Nonidentifiable risk factor	30 (16.7)	9 (13.4)	21 (18.6)	.37
Obesity	72 (40.0)	25 (37.3)	47 (41.6)	.57
Major surgery	12 (6.7)	5 (7.5)	7 (6.2)	.74
Immobilization	79 (43.9)	31 (46.3)	48 (42.5)	.62
Estrogen use	3 (1.7)	3 (4.5)	0 (0.0)	.02
Long-distance travel	1 (0.6)	1 (1.5)	0 (0.0)	.19
Autoimmune disease	20 (11.1)	8 (11.9)	12 (10.6)	.79
Hereditary thrombophilia	2 (1.1)	1 (1.5)	1 (0.9)	.71
Active malignancy	37 (20.6)	14 (20.9)	23 (20.4)	.93
Lower extremity fracture	9 (5.0)	3 (4.5)	6 (5.3)	.80
Pregnancy or postpartum	0 (0.0)	0 (0.0)	0 (0.0)	-
Treatment				
Deny treatment	2 (1.1)	1 (1.5)	1 (0.9)	.71
Anticoagulants				
LMWH alone	46 (25.6)	16 (23.9)	30 (26.6)	.69
LMWH followed by VKA	85 (47.2)	31 (46.3)	54 (47.8)	.84
Direct oral anticoagulants	42 (23.3)	17 (25.4)	25 (22.1)	.62
IVC filter	4 (2.2)	1 (1.5)	3 (2.7)	.61
Endovascular stent	1 (0.6)	1 (1.5)	0 (0.0)	.19

Data are presented as *n* (%).

CT, computed tomography; ICS, iliac compression syndrome; IVC, inferior vena cava; LMWH, low-molecular-weight heparin; MR, magnetic resonance; MRI, magnetic resonance imaging; VKA, vitamin K antagonist.

### 3.3 ICS

Among the 180 patients, 67 had ICS, with a prevalence of 37.2%. The median age in the ICS group was lower than that in the non-ICS group (58 vs 71 years; *P* < .001). The prevalence did not differ significantly between males and females (64.2% vs 35.8%; *P* = .86). Left leg DVT was more frequent in the ICS group than in the non-ICS group (62.7% vs 23.9%; *P* = .01), whereas in the non-ICS group, right and left leg DVT occurred equally (43.4%). The iliac vein was a common site of DVT in the ICS group, whereas the femoropopliteal vein was frequently involved in the non-ICS group. There was no statistically significant difference in iliac vein involvement (34.3% vs 22.1%; *P* = .07) between the 2 groups. ICS was diagnosed with an abdominal CT scan, CT angiography, abdominal MRI, and MR angiography (68.7%, 23.9%, 6%, and 1.5%, respectively). Estrogen use was more frequent in the ICS group than in the non-ICS group (4.5% vs 0%; *P* = .02). Endovascular stenting was performed in 1.5% of ICS patients. The percentage of mean iliac vein compression was 62.4% and 27.9% in the ICS and non-ICS groups, respectively. There was a significant difference in the mean iliac vein compression percentage between the 2 groups (*P* < .001).

Univariate logistic regression revealed that age, BMI, and iliac vein involvement were associated with ICS. However, when multivariate logistic regression was performed to identify factors associated with ICS, only age was significantly associated with ICS (OR, 0.96; 95% CI, 0.94-0.98). When age was categorized into groups, individuals aged <30 years showed the strongest association with ICS (OR, 17.37; 95% CI, 2.14-140.5; *P* = .007).

### 3.4 Outcomes

Seventeen patients (9.4%) experienced recurrent DVT. Among them, 10 (58.8%) developed recurrence while receiving anticoagulant therapy, with a median time to recurrence of 2.59 years (IQR, 0.34-4.94). The remaining 7 patients (41.2%) developed recurrence after discontinuation of anticoagulants, with a median time to recurrence of 0.50 years (IQR, 0.39-2.06). Recurrent DVT was significantly higher in the ICS group than in the non-ICS group (17.9% vs 4.4%; OR, 4.71; 95% CI, 1.6-14.1; *P* = .003). There was no difference in PE between the 2 groups (13.4% vs 20.4%; OR, 0.61; 95% CI, 0.3-1.4; *P* = .24). The percentage of PTS in the ICS group was numerically higher than that in the non-ICS group, but there was no significant difference between the 2 groups (4.5% vs 1.8%; OR, 2.6; 95% CI, 0.4-16; *P* = .3; Table 2).

**Table 2:** Recurrent deep vein thrombosis, pulmonary embolism, and postthrombotic syndrome in patients with iliac compression syndrome.

Outcomes	All patients (n = 180)	ICS (*n* = 67)	Non-ICS (*n* = 113)	OR (95% CI)	*P* value
Recurrent DVT	17 (9.4)	12 (17.9)	5 (4.4)	4.71 (1.6-14.1)	.003
PE	32 (17.8)	9 (13.4)	23 (20.4)	0.61 (0.3-1.4)	.24
PTS	5 (2.8)	3 (4.5)	2 (1.8)	2.60 (0.4-16.0)	.30

Data are presented as *n* (%).

DVT, deep vein thrombosis; ICS, iliac compression syndrome; OR, odds ratio; PE, pulmonary embolism; PTS, postthrombotic syndrome.

Univariate logistic regression for recurrent DVT was performed to identify candidate variables for multivariate analysis. Age, the presence of ICS, prior venous thromboembolism (VTE), and underlying autoimmune diseases were included in the multivariate logistic regression. ICS (OR, 8.26; 95% CI, 2.03-33.66; *P* = .003), previous VTE (OR, 34.29; 95% CI, 7.38-159.1; *P* < .001), and autoimmune diseases (OR, 6.31; 95% CI, 1.47-26.96; *P* = .01) were significantly associated with recurrent DVT.

## 4 Discussion

In our study, we reviewed imaging of patients diagnosed with DVT and found that the prevalence of ICS was 37%. Notably, younger age was significantly associated with ICS, with a strong association observed in individuals aged <30 years. Recurrent DVT was significantly higher in the ICS group than in the non-ICS group. There was no difference in PE or PTS between ICS patients and non-ICS patients. We found that ICS, previous VTE, and underlying autoimmune diseases were independently associated with recurrent DVT.

Compression of the iliac vein by >50% has been reported in approximately 10% to 37% of asymptomatic individuals, suggesting that such compression may represent a physiological variant rather than a definitive risk factor for DVT [[8], [9], [10]]. However, studies specifically evaluating ICS in DVT populations remain limited. A study from China reported that 58% of patients with DVT who underwent imaging had compression of the CIV exceeding 50%. Notably, this study excluded patients with bilateral or right-sided DVT, potentially introducing selection bias by focusing on a population more likely to have ICS [11]. In our study, we observed a prevalence of ICS comparable to that reported previously [1,2]. Another study of DVT patients demonstrated a lower incidence of PE in those with ICS, defined as iliac vein compression >50%, compared with non-ICS patients [12]. Although the difference in PE incidence between the ICS and non-ICS groups in our cohort was not statistically significant, the proportion of PE was slightly lower in the ICS group (13.4% vs 20.4%). The mean iliac vein compression reported in that study was 42.9% [12], which is comparable to our finding of 40.7%.

ICS was initially described as chronic iliac vein compression leading to endothelial irritation and intraluminal spur formation. Early definitions relied on evidence of iliac vein thrombosis and compression seen on venography or intravascular ultrasound, or the presence of collateral vessels. However, these imaging modalities are invasive and not routinely performed. More recently, a threshold of >50% iliac vein compression on cross-sectional imaging has been proposed as a practical diagnostic criterion. A study of 500 asymptomatic individuals demonstrated that ≥50% iliac vein compression was associated with an increased risk of DVT [13]. In our study, we adopted this definition in line with the published literature. Still, the lack of a standardized definition highlights the need for further research to establish consistent diagnostic criteria for ICS.

While previous studies have identified factors such as female sex, scoliosis, dehydration, hypercoagulable disorders, and cumulative radiation exposure associated with ICS, our study found that age was a significant factor. The strong association between ICS and DVT in individuals aged <30 years underscores the need for heightened clinical awareness in younger populations presenting with DVT. We suggest that patients younger than 30 years with iliofemoral DVT be considered for further CT imaging to assess for ICS, particularly those with recurrence and no identifiable risk factor.

Despite detecting ICS in 37% of patients, only 6 (3.3%) were initially suspected of having ICS from imaging reports. This discrepancy suggests underrecognition of ICS in clinical practice, potentially due to variability in imaging indications and reporting. Many imaging studies in our cohort were performed in the context of cancer screening, which may have limited detailed assessment of iliac vein compression.

Our multivariate analysis identified ICS, previous VTE, and underlying autoimmune diseases as independent risk factors for recurrent DVT. These findings are consistent with existing literature, which recognizes prior VTE and autoimmune conditions as established contributors to recurrence [14]. While recurrent DVT in the context of ICS has been reported previously, most of the evidence comes from case reports. To our knowledge, this is the first study to demonstrate that ICS is an independent risk factor for recurrent DVT in a cohort-based analysis.

This study has several strengths. First, we conducted a focused evaluation of ICS prevalence in a real-world DVT cohort, an area that remains underexplored. Second, imaging studies of all eligible patients were systematically reviewed. Third, all imaging studies were independently reviewed by 2 radiologists, enhancing diagnostic reliability.

However, there are some limitations. The data were limited due to the single-center design and its retrospective nature. The documentation of PTS symptoms was incomplete, which may have led to underreporting of its incidence. The sample size calculated in our study aimed to determine the prevalence. Therefore, it might have lacked sufficient power to detect a difference in the outcome. Additionally, selection bias is a concern, as not all DVT patients underwent abdominal CT scans or MRI. However, this limitation may be mitigated by the well-balanced baseline characteristics, such as sex, affected leg, and presenting symptoms, of the ICS and non-ICS groups.

## 5 Conclusion

The prevalence of ICS in DVT patients in this study was 37.2%. Younger age was significantly associated with ICS. ICS, previous VTE, and autoimmune disease were associated with an increased risk of recurrent DVT. ICS might be an underinvestigated contributor to recurrent DVT and warrants further exploration in prospective studies.
